# Effects of a school-based ACT program on motivation, physical activity participation, motor competence, and physical performance in physically inactive female adolescent students

**DOI:** 10.1371/journal.pone.0343227

**Published:** 2026-06-10

**Authors:** Hassan Gharayagh Zandi, Fatemeh Shaabani, Azam Noferesti, Elaheh Arab Ameri

**Affiliations:** 1 Department of Behavioral and Cognitive Sciences in Sports, Sports and Health Sciences Faculty, University of Tehran, Tehran, Iran; 2 Department of Psychology, Faculty of Educational Sciences and Psychology, University of Tehran, Tehran, Iran; University of Tartu, ESTONIA

## Abstract

Adolescents face internal barriers like low self-confidence and psychological discomfort that limit physical activity. Adolescence offers a key window to enhance intrinsic motivation. Acceptance and Commitment Therapy (ACT) may effectively improve physical activity and motor competence through psychological flexibility and value-based behavior. This study examined the effects of an ACT-based intervention on physical activity participation, motivation, motor competence, and physical fitness in physically inactive adolescent girls. A randomized controlled trial involved 132 inactive girls aged 13–18, randomly assigned to either an ACT intervention or a control group. Intervention included seven sessions targeting six core ACT processes. Pre-and post-intervention assessments used standardized questionnaires and tests for physical activity participation, motivation, physical activity, motor competence, and fitness. ACT group showed significant improvements in intrinsic motivation, identified regulation, autonomy, and reduced amotivation and external regulation (p ≤ 0.05). Introjected regulation did not show a significant change (p > 0.05). Physical activity participation and fitness performance also improved significantly, along with both perceived and actual motor competence (p ≤ 0.05). ACT-based intervention effectively enhanced motivation, physical activity engagement, motor competence, and fitness. These findings support integrating ACT into school or community programs to promote sustainable physical activity habits in adolescent girls.

## Introduction

Adolescence is a critical period for establishing lifelong physical activity habits. Yet, over 80% of adolescents worldwide fail to meet the WHO recommendation of 60 minutes of daily moderate-to-vigorous physical activity, placing them at increased risk for obesity, metabolic disorders, and mental health issues [1,2]. Traditional interventions often rely on extrinsic motivators or skill-based approaches, which may produce only short-term improvements [3,4]. However, such programs frequently overlook internal psychological barriers—like low self-efficacy, fear of judgment, and experiential avoidance—that prevent inactive adolescents from sustaining regular physical activity [5,6].

To address these internal barriers, Acceptance and Commitment Therapy (ACT) offers a promising approach. ACT targets six core processes: acceptance, cognitive defusion, mindfulness, self-as-context, values clarification, and committed action [7–9]. By enhancing psychological flexibility—the capacity to persist in valued behaviors despite unpleasant thoughts or emotions—ACT provides adolescents with strategies to engage in physical activity even in the presence of internal or emotional obstacles [8,10]. This aligns closely with Self-Determination Theory (SDT), as value-congruent actions can strengthen autonomous motivation, perceived competence, and long-term adherence [9,11]. Recent research by Ahmadi et al. (2023) further supports this integration, suggesting that ACT can serve as a theory-based, SDT-informed method for enhancing motivation in adolescents [12].

Alth Despite ACT’s demonstrated effectiveness in adults and college students [13–15], research in adolescents is limited. Existing studies often involve small samples, lack standardized protocols, and focus on narrow outcomes, neglecting comprehensive measures such as motor competence and physical performance [8,10,13,14,16]. Notably, no randomized trial has examined whether a school-based ACT program can simultaneously improve motivation, physical activity, motor skills, and fitness in physically inactive adolescent girls—a population particularly at risk for activity decline during this developmental stage [3,17,18].

Schools provide an ideal setting for interventions, offering equitable access across socioeconomic groups and integration with physical education curricula [19,20]. Given these advantages, we designed a seven-session, theory-driven ACT program to target motivational regulation, physical activity participation, motor competence, and physical performance in inactive female adolescents. We hypothesize that adolescents who receive the ACT-based intervention will show significantly greater improvements in motivation, motor competence, and physical performance compared to a control group. Therefore, the present study aims to evaluate the effects of an ACT-based intervention on physical activity participation, motivation, motor competence, and physical performance in physically inactive adolescents.

## Materials and methods

### Study design

**Fig 1:**
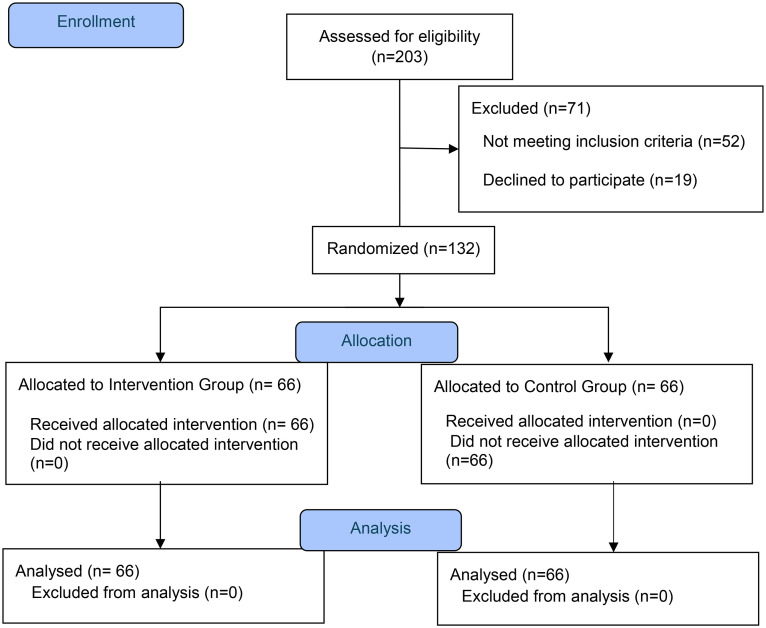
CONSORT flowchart of the study deign.

This parallel-group randomized controlled trial with a repeated-measures design aimed to examine the longitudinal effects of an ACT intervention on motivation, physical activity participation, motor competence, and physical performance in physically inactive female adolescent students. The study was conducted in Shahrood County during the 2024–2025 academic year. Participants were randomly assigned to either the intervention or a wait-list control group (Fig 1). Ethical approval was obtained from the Ethics Committee of Shahrood University of Technology (IR.SHAHROODUT.REC.1403.019), and the trial was registered in the University Hospital Medical Information Network Clinical Trial Registry under registration number UMIN000057926 on 2025/05/21.

### Recruitment and participants

Based on prior research [10], the required sample size was calculated using G*Power to achieve 85% power at a 95% confidence level, resulting in a minimum of 102 participants. To account for an anticipated 20% dropout rate, 132 female students were recruited via convenience sampling from eight public girls’ schools in Shahrood, Semnan, Iran. Following approval from the Faculty of Physical Education and Sport Sciences – University of Tehran, coordination with school principals, and briefing sessions with parents and students, eligible participants were selected based on predefined inclusion criteria. Participant recruitment took place between September 22, 2024, and October 17, 2024, with follow-up assessments completed by April 20, 2025. The recruitment period aligned with the 2024–2025 academic year to correspond with the school calendar. Inclusion criteria required female students aged 13–18 years who were physically able to safely participate in physical activities, as confirmed by the Physical Activity Readiness Questionnaire (PAR-Q) and clinical evaluation. Additionally, participants needed to engage in less than 60 minutes of moderate-intensity physical activity daily, indicated by a PAQ-A score below 2.75 [21]. Exclusion criteria included chest pain during activity, musculoskeletal limitations, use of specific or psychoactive medications, diagnosed mental or neurological disorders, or unwillingness to participate.

All participants and parents provided written informed consent after being informed about the study’s purpose and procedures, were assured of confidentiality and their right to withdraw at any time, and all procedures were conducted in accordance with the principles of the Declaration of Helsinki.

### Randomization

A stratified cluster randomization approach was used to allocate 8 schools into Acceptance and Commitment Therapy (ACT) group (n = 66) or the wait-list control group (n = 66). Schools were first stratified by educational level—lower secondary (grades 7–9) and upper secondary (grades 10–12). Within each stratum, random numbers were generated using SPSS, and schools were ranked accordingly. Two schools from each stratum were then randomly assigned to the intervention group and two to the wait-list control group, ensuring balanced representation and minimizing group contamination. The random allocation sequence was created and securely maintained by an independent statistician, with study staff involved in recruitment and data collection blinded to group assignments until allocation was complete. School principals facilitated participant enrollment based on the assigned groups. Outcome assessors and data analysts were blinded throughout the study to reduce bias, while blinding of participants and school staff was not feasible due to the intervention’s nature.

### Intervention condition (ACT protocol)

Participants in the intervention group took part in a structured ACT program. The program was designed to enhance psychological flexibility by targeting six core processes: willingness and acceptance, mindfulness, cognitive defusion, self-as-context, values clarification, and committed action. This protocol was developed based on validated prior studies [7–10,16,22], with further details provided in Table 1. The program consisted of seven weekly sessions, each lasting 45–60 minutes, conducted in person in a quiet meeting room at the school. Participants were divided into four separate groups, each consisting of 16 or 17 individuals. The sessions were led by a single sport psychology therapist experienced in ACT interventions (FS, the researcher of this study). To ensure fidelity and consistent delivery across groups, the therapist received comprehensive pre-intervention training and ongoing supervision throughout the program by an experienced clinical psychologist (AN, the researcher of this study). To ensure intervention fidelity, a structured fidelity checklist was developed based on the six core ACT processes. The checklist items assessed adherence to session content, accurate delivery of ACT components, therapist competence, and participant engagement. All sessions demonstrated at least 96% adherence to the protocol, with a mean adherence of 98.3%. Additionally, weekly supervision meetings between the therapist and the clinical psychologist (AN) were conducted to maintain consistency and promptly address any deviations. Each session included psychoeducation, the use of metaphors, experiential exercises, and homework assignments aimed at facilitating skill practice between sessions. A brief overview of each session’s content is provided in Table 1, summarizing key activities and therapeutic focuses. Participants’ progress was monitored through systematic review of homework assignments and encouraging active participation in group discussions. We evaluated progress based on qualitative feedback and participant engagement, not on standardized quantitative measures. All sessions were held regularly according to a predetermined weekly schedule.

**Table 1:** Overview of the seven-session Acceptance and Commitment Therapy (ACT) intervention program.

Session	Goal	Topics Covered
**1: Introduction**	Introduce the program structure and the importance of physical activity for well-being.	- Introduction to the program - Why physical activity matters - Relational Frame Theory (RFT) framework - Relationship between language and mind
**2: Acceptance**	Help participants understand experiential avoidance and encourage embracing thoughts and feelings.	- What is experiential avoidance? - Why is overcoming experiential avoidance important? - Metaphors for experiential avoidance - Identifying avoidance thoughts and emotions - Constructive despair - Metaphor: “ The Well” and “ The Unwelcome Guest”
**3: Cognitive Defusion**	Teach participants to detach from their thoughts and view them without judgment.	- What is cognitive fusion? - Metaphor: “ Teflon Container” - What is cognitive defusion? - Metaphor: “ Bus Passengers” - Exercises: “ Floating Leaves” and “ Milk Carton”
**4: Mindfulness**	Introduce mindfulness techniques to enhance present-moment awareness and clarity.	- What is mindfulness? - Why live in the moment? - Exercises: Mindful walking, “ Mindful Tape,” “ Hand Exercise”
**5: Self-as-Context**	Help participants see themselves from a broader perspective, reducing attachment to thoughts and emotions.	- Self as context vs. self as content - Benefits of self as context - Metaphor: “ King and Shepherd” - Metaphor: “ Chessboard” - Exercise: “ Speaking and Listening”
**6: Values Clarification**	Help participants identify core values and use them to guide meaningful actions.	- What are values? - Why identify your values? - Metaphor: “ Bus Passengers” - Values for physical education students
**7: Committed Action**	Teach participants to set action plans aligned with their values, overcome obstacles, and seek support.	- How to choose goals that reflect your values - Setting SMART goals - Taking small steps - Anticipating obstacles - Using positive reinforcement - Seeking support - Using reminders

### Wait-list control condition

Participants in the wait-list control group did not receive any intervention during the study period. This group was comparable in size and demographic characteristics to the intervention group (exact numbers and details provided in Table 2). Follow-up assessments were conducted at the same time points as for the intervention group to allow for valid comparisons. After completing the follow-up assessments, the same ACT program was offered to participants in the wait-list control group. During the study period, participants in the wait-list group did not receive any other psychological or behavioral interventions and were asked to maintain their usual routines without additional treatment or contact with the therapists.

**Table 2:** Demographic and anthropometric characteristics of participants in the intervention and control groups (N = 132).

Variables	Intervention Group (n = 66)	Control Group (n = 66)
**Age (years)**	15.5 ± 1.7	15.5 ± 1.7
**Height (cm)**	159.1 ± 6.6	158.4 ± 6.1
**Weight (kg)**	54.5 ± 9.7	55.1 ± 9.2
**Body Mass Index (kg/m²)**	21.5 ± 3.2	21.8 ± 2.6

***Note*.** Values are presented as Mean ± SD.

### Outcome measures

The research team conducted all assessments at three key points: prior to the intervention (October 2024), immediately following the seven-week Acceptance and Commitment Therapy (ACT) program (December 2024), and during a three-month follow-up (March 2025).

- **Motivation for Physical Activity:** Motivation was measured using the Behavioral Regulation in Exercise Questionnaire-2 (BREQ-2), which contains 19 items assessing different motivational types: intrinsic regulation (4 items, e.g., “I exercise because it is an enjoyable activity”), identified regulation (4 items, e.g., “I value the benefits of exercise”), introjected regulation (3 items, e.g., “I feel guilty when I don’t exercise”), external regulation (4 items, e.g., “I exercise because others tell me to”), and amotivation (4 items, e.g., “I don’t know why I should exercise”). Participants rated items on a 5-point Likert scale from 0 (not true for me) to 4 (completely true for me). Subscale scores were calculated as the mean of corresponding items. A Relative Autonomy Index (RAI) was derived by weighting each subscale (intrinsic = 3, identified = 2, introjected = −1, external = −2, amotivation = −3) and summing the results, with possible scores ranging from −24–20; higher scores reflect greater autonomous motivation [23]. The Persian version demonstrates acceptable internal consistency (α > 0.7) and high test-retest reliability (ICC > 0.80) [24].
- **Physical Activity Participation:** The Physical Activity Questionnaire for Adolescents (PAQ-A) assessed average participation in physical and sports activities over the past week. It includes 8 items, with the first listing 22 physical/sports activities plus an “other” option; responses are scored from 1 to 5 based on frequency. Remaining items use a 5-point Likert scale ranging from low (1) to high (5), and the mean of all items yields the overall PAQ score. An additional question screens for illness or activity restrictions in the prior week but is not included in the total score [21]. The Persian PAQ-A has demonstrated strong reliability (Cronbach’s alpha = 0.89) and validated content and face validity through expert review [25].
- **Physical Performance:** Health-related fitness components were evaluated using a battery of standardized tests: The standing long jump measured lower-body explosive strength by recording the distance participants jumped forward from a standing start. Speed was evaluated with a 20-meter sprint, timing participants as they ran at maximum effort. Cardiovascular endurance was gauged using the Cooper 6-minute run/walk test, which recorded the distance covered in six minutes of continuous movement. Flexibility was assessed via the sit-and-reach test, where participants reached forward while seated with legs extended, and the maximum reach distance was recorded [26,27].
- **Perceived Motor Competence:** Evaluated by the Adolescent Motor Competence Questionnaire (AMCQ), a self-report measure designed for adolescents aged 12–18 years. The AMCQ assesses motor skills and daily functional activities aligned with DSM-V criteria and Developmental Coordination Disorder (DCD) guidelines. It consists of items scored on a 4-point Likert scale from “never” (1) to “always” (4), with negatively phrased questions reverse-scored to reduce bias. Maximum scores reach 104, with values ≤ 83 indicating potential motor difficulties [28]. The AMCQ’s validity was confirmed against the McCarron Neurodevelopmental Assessment (MAND), and it shows excellent reliability (test-retest ICC = 0.956; internal consistency α = 0.902). The Persian version similarly demonstrates excellent content validity (CVI = 0.980), internal consistency (α = 0.940), and test-retest reliability (ICC = 0.885) [29].
- **Actual Motor Competence:** Assessed through the Motor Competence Test (TMC), comprising four tasks measuring fine and gross motor skills: two fine motor tasks—placing bricks on a board (PB) and building a brick tower (BB)—and two gross motor tasks—heel-to-toe walking (HTW) and walking/running in a figure-eight pattern (W/R). The PB task requires placing 18 Duplo bricks on a 3x6 board as quickly as possible; the BB task involves building a tower with 12 bricks without touching the table. The HTW task involves walking 4.5 meters heel-to-toe in a straight line, while the W/R task requires walking or running a figure-eight pattern [30]. The TMC shows good reliability (above 0.6 across age groups) and strong construct validity (CFI and TLI > 0.9, RMSEA > 0.08) within the Iranian population [31].

### Statistical analyses

Data were analyzed using SPSS version 21. Descriptive and inferential statistical methods were employed. The normality of data distribution was assessed using the Shapiro–Wilk test (preferred for sample sizes < 50 per group; note: Kolmogorov–Smirnov is less powerful and generally discouraged), and Levene’s test was used to evaluate homogeneity of variances. To test the research hypotheses, a 2 × 3 mixed-design analysis of variance (ANOVA) was conducted, with group (intervention group based on Acceptance and Commitment Therapy and wait-list control group) as the between-subjects factor and time (pre-test, post-test, and 3-month follow-up) as the within-subjects factor. The analysis examined both main effects and the group × time interaction effect for all outcome variables. Partial eta squared (η²p) values will be reported as an effect size indicator, with values of 0.01–0.059, 0.06–0.139, and >0.14 indicating small, medium, and large effects, respectively. Follow-up pairwise comparisons were conducted using Bonferroni-corrected post hoc tests to examine within- and between-group differences across time points. All evaluations will be conducted at a significance level of α = 0.05.

## Results

A total of 132 participants and their parents provided informed consent to participate in the study. All participants completed the questionnaires and tests at baseline, post-test, and follow-up. Adherence to the intervention was very high, with participants attending 98.3% of all scheduled educational sessions and completing all planned testing procedures as intended (Fig 1).

Table 2 shows that the intervention and wait-list control groups were comparable at baseline in terms of age, height, weight, and body mass index (BMI). The mean age was 15.5 years (SD = 1.7) in both groups. Height was similar across groups, with the intervention group averaging 159.1 cm (SD = 6.6) and the control group 158.4 cm (SD = 6.1). The mean weight was slightly lower in the intervention group (M = 54.5 kg, SD = 9.7) compared to the control group (M = 55.1 kg, SD = 9.2). BMI was also slightly lower in the intervention group (M = 21.5, SD = 3.2) than in the control group (M = 21.8, SD = 2.6). These results suggest that the groups were well balanced prior to the intervention.

Results indicated significant time × group interaction effects for amotivation, *F*(2, 260) = 21.08, *p* =.001, ηp² =.14; external regulation, *F*(2, 260) = 4.45, *p* =.01, ηp² =.03; identified regulation, *F*(2, 260) = 9.17, *p* =.001, ηp² =.06; intrinsic regulation, *F*(2, 260) = 45.31, *p* =.001, ηp² =.25; and autonomy index, *F*(2, 260) = 26.36, *p* =.001, ηp² =.16. No significant interaction effect was found for introjected regulation, *F*(2, 260) = 0.83, *p* =.43, ηp² =.006. Effect size estimates suggest that the ACT intervention had the strongest impact on intrinsic regulation (25%), amotivation (14%), and the autonomy index (16%), with smaller but significant effects observed for identified regulation (6%) and external regulation (3%). These findings indicate that the ACT intervention was effective in enhancing intrinsic motivation and autonomy while reducing amotivation among participants (Table 3).

**Table 3:** Means and standard deviations for motivation, physical activity, motor competence, and physical fitness variables by group and time point, with repeated measures ANOVA results.

Variables	Total (n = 132)	Intervention Group (n = 66)	Control Group (n = 66)	Repeated measure ANOVA		
				Time	Group	Time × Group
Amotivation (0–4)						
**Pre-test**	0.92 ± 0.66	0.94 ± 0.67	0.89 ± 0.64	0.001	0.06	0.001
**Post-test**	0.56 ± 0.50	0.42 ± 0.38	0.71 ± 0.57			
**Follow-up**	0.59 ± 0.51	0.44 ± 0.39	0.75 ± 0.54			
External Regulation (0–4)						
**Pre-test**	0.92 ± 0.73	0.89 ± 0.74	0.95 ± 0.72	0.001	0.06	0.01
**Post-test**	0.59 ± 0.51	0.45 ± 0.41	0.73 ± 0.67			
**Follow-up**	0.60 ± 0.56	0.49 ± 0.44	0.72 ± 0.67			
Introjected Regulation (0–4)						
**Pre-test**	1.72 ± 0.86	1.62 ± 0.84	1.82 ± 0.88	0.001	0.03	0.43
**Post-test**	1.54 ± 0.86	1.38 ± 0.94	1.71 ± 0.75			
**Follow-up**	1.54 ± 0.86	1.38 ± 0.94	1.70 ± 0.76			
Identified Regulation (0–4)						
**Pre-test**	2.50 ± 0.70	2.53 ± 0.63	2.60 ± 0.77	0.001	0.28	0.001
**Post-test**	2.72 ± 0.71	2.79 ± 0.62	2.64 ± 0.78			
**Follow-up**	2.72 ± 0.73	2.86 ± 0.62	2.58 ± 0.80			
Intrinsic Motivation (0–4)						
**Pre-test**	2.83 ± 0.63	2.89 ± 0.61	2.78 ± 0.65	0.001	0.001	0.001
**Post-test**	3.09 ± 0.67	3.34 ± 0.59	2.83 ± 0.64			
**Follow-up**	3.09 ± 0.69	3.36 ± 0.61	2.82 ± 0.65			
Autonomy Index						
**Pre-test**	7.32 ± 4.11	7.50 ± 4.00	7.14 ± 2.24	0.001	0.003	0.001
**Post-test**	10.30 ± 4.00	12.10 ± 3.08	8.50 ± 4.02			
**Follow-up**						
Physical Activity Participation (1–5)						
**Pre-test**	1.97 ± 0.43	1.98 ± 0.43	1.95 ± 0.40	0.001	0.001	0.001
**Post-test**	2.14 ± 0.50	2.33 ± 0.48	1.94 ± 0.45			
**Follow-up**	2.21 ± 0.54	2.42 ± 0.55	1.98 ± 0.44			
Heel-to-Toe (seconds)						
**Pre-test**	10.72 ± 2.01	10.90 ± 2.00	10.54 ± 2.02	0.001	0.21	0.001
**Post-test**	9.74 ± 1.70	9.30 ± 1.42	10.19 ± 1.84			
**Follow-up**	9.67 ± 1.67	9.19 ± 1.47	10.14 ± 1.74			
Walking/Running (seconds)						
**Pre-test**	6.58 ± 0.58	6.17 ± 0.60	6.00 ± 0.54	0.44	0.001	0.001
**Post-test**	5.70 ± 0.61	5.55 ± 0.56	5.85 ± 0.62			
**Follow-up**	5.70 ± 0.61	5.48 ± 0.48	5.92 ± 0.63			
Placing on the Board (seconds)						
**Pre-test**	23.63 ± 3.92	23.04 ± 3.71	23.83 ± 3.93	0.001	0.004	0.001
**Post-test**	21.84 ± 3.85	19.12 ± 3.16	20.21 ± 3.11			
**Follow-up**	21.95 ± 3.86	20.63 ± 3.43	23.27 ± 3.84			
Building a Tower (seconds)						
**Pre-test**	13.77 ± 2.77	13.28 ± 2.62	13.34 ± 2.93	0.001	0.01	0.001
**Post-test**	11.92 ± 2.64	11.35 ± 2.19	12.65 ± 2.72			
**Follow-up**	11.90 ± 2.62	11.02 ± 2.28	12.79 ± 2.65			
Perceived Motor Competence (1–4)						
**Pre-test**	88.55 ± 5.82	88.22 ± 5.57	88.87 ± 6.08	0.001	0.04	0.001
**Post-test**	91.62 ± 5.39	92.45 ± 4.80	90.34 ± 5.34			
**Follow-up**	91.11 ± 5.53	93.18 ± 4.68	89.04 ± 5.58			
Cooper (km)						
**Pre-test**	974.30 ± 131.38	952.40 ± 106.95	996.59 ± 150.27	0.001	0.01	0.001
**Post-test**	1081.44 ± 123.49	1131.03 ± 94.94	1031.84 ± 129.32			
**Follow-up**	1107.68 ± 131.47	93.02 ± 1177.15	127.98 ± 1038.21			
Flexibility (cm)						
**Pre-test**	25.19 ± 6.49	24.92 ± 6.00	25.46 ± 6.99	0.001	0.2	0.001
**Post-test**	28.70 ± 5.76	29.56 ± 5.18	27.84 ± 6.21			
**Follow-up**	29.25 ± 5.66	5.18 ± 30.57	5.85 ± 27.92			
Jump (meters)						
**Pre-test**	151.90 ± 16.73	150.87 ± 14.80	152.12 ± 19.08	0.001	0.37	0.001
**Post-test**	158.26 ± 18.10	160.71 ± 17.00	156.30 ± 18.86			
**Follow-up**	159.34 ± 17.90	16.32 ± 162.5	18.60 ± 156.51			
20-Meter Run (sec)						
**Pre-test**	4.56 ± 0.56	4.65 ± 0.50	4.47 ± 0.60	0.001	0.03	0.001
**Post-test**	4.25 ± 0.59	4.05 ± 0.55	4.45 ± 0.57			
**Follow-up**	4.27 ± 0.55	4.50 ± 0.07	4.53 ± 0.48			

**Fig 2:**
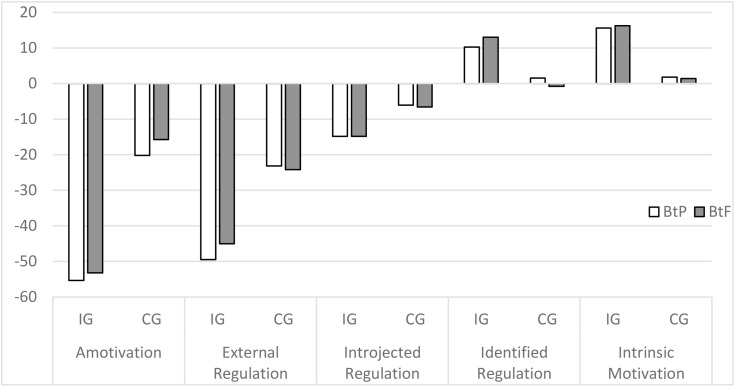
Percentage changes in motivational patterns by group from Baseline to Posttest (BtP) and Baseline to Follow-up (BtF).

The results of the Bonferroni test indicated that the intervention group showed significantly greater improvements in motivational patterns compared to the control group. Specifically, amotivation decreased by 55.32% in the intervention group versus 20.22% in the control group at post-test (p <.001), and by 53.19% versus 15.73% at follow-up (p <.001). External regulation also declined more in the intervention group (49.44% vs. 23.16%, p =.007 at post-test; 45.00% vs. 24.21%, p =.02 at follow-up). Introjected regulation decreased by 14.81% in the intervention group compared to 6.04% in the control group at post-test (p =.03), and by a similar pattern at follow-up (14.81% vs. 6.59%, p =.04). Identified regulation improved more in the intervention group than in the control group at post-test (10.28% vs. 1.54%), although the difference was not statistically significant (p =.20); however, a significant difference emerged at follow-up (13.04% vs. a 0.77% decline, p =.02). Finally, intrinsic motivation increased by 15.57% at post-test and 16.26% at follow-up in the intervention group, while the control group showed only minimal changes (1.79% and 1.44%, respectively; both p <.001; see Fig 2).

Significant time × group interaction effects were observed for physical activity participation, F(2, 260) = 21.08, p =.001, ηp² =.14, indicating a large intervention effect on changes in physical activity over time. Similarly, significant interaction effects were found for the Cooper test (F(2, N) = 19.36, p <.001, ηp² =.13), flexibility (F(2, N) = 23.27, p <.001, ηp² =.15), standing long jump (F(2, N) = 12.14, p <.001, ηp² =.08), and 20-meter run (F(2, N) = 53.29, p <.001, ηp² =.29). Effect sizes indicate that the ACT intervention had the greatest impact on the 20-meter run (29%), followed by flexibility (15%), the Cooper test (13%), and long jump (8%), suggesting significant improvements in physical performance across multiple tasks compared to the control group (Table 3).

**Fig 3:**
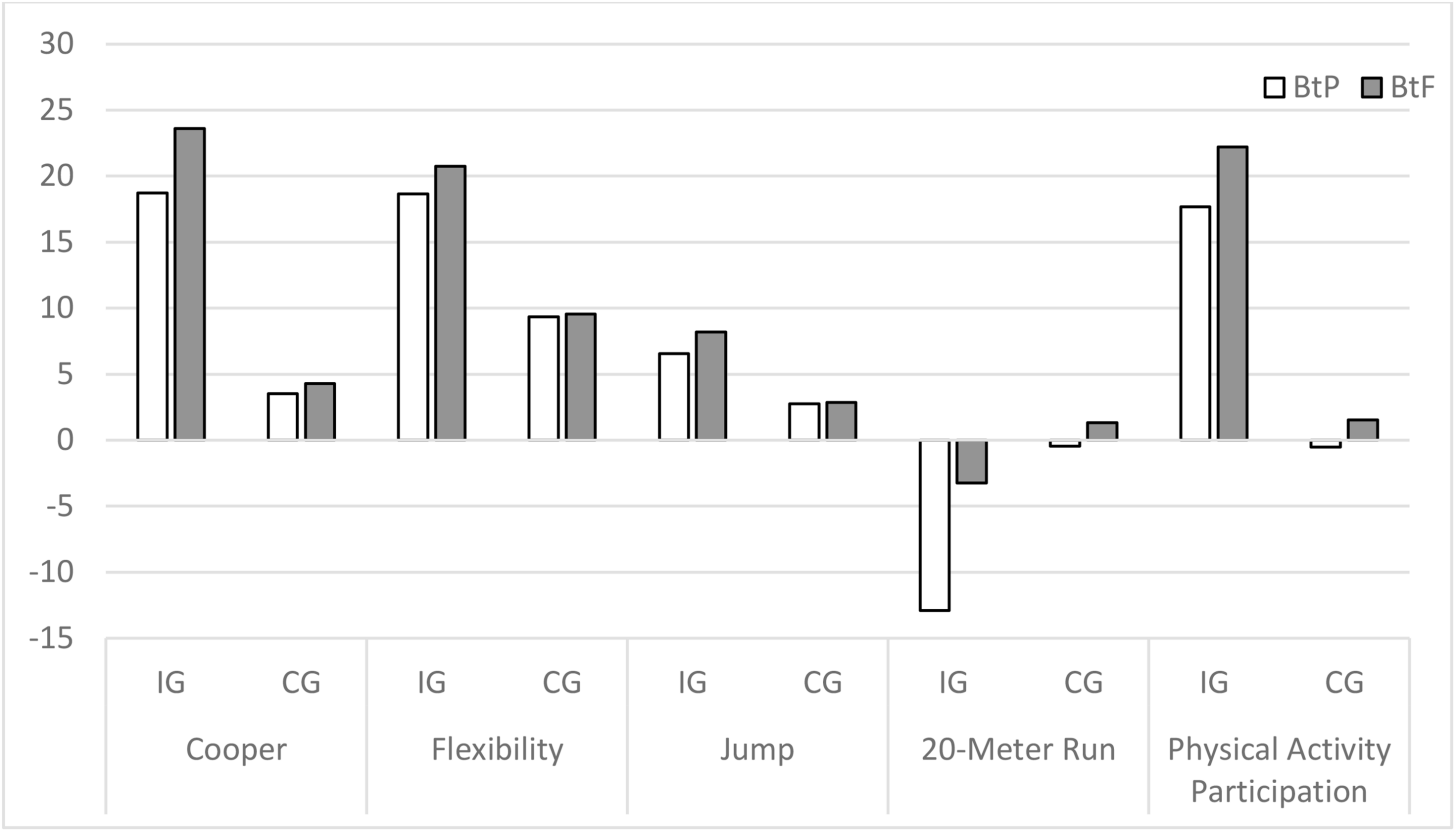
Percentage changes in physical activity participation and physical fitness by group from Baseline to Posttest (BtP) and Baseline to Follow-up (BtF).

Bonferroni post hoc analyses showed that the intervention group experienced significantly greater increases in physical activity participation—17.68% at post-test and 22.2% at follow-up—compared to minimal changes in the control group (−0.51% and 1.54%, respectively; p <.001). The intervention group also showed marked improvements in the Cooper test at post-test (18.74% vs. 3.53%) and follow-up (23.59% vs. 4.28%; p <.001). Flexibility improved by 18.66% at post-test (p =.053) and 20.75% at follow-up (p =.003) in the intervention group, compared to 9.33% and 9.57% in controls. Although the difference in standing long jump at post-test was not significant (6.54% vs. 2.77%; p =.12), the intervention group showed significantly greater gains at follow-up (8.2% vs. 2.87%; p =.04). Finally, the 20-meter run time decreased significantly in the intervention group at post-test (−12.9% vs. −0.45%) and follow-up (−3.23% vs. 1.34%; p <.001; see Fig 3), indicating enhanced speed and cardiovascular efficiency following the ACT intervention.

The analysis revealed significant time × group interaction effects for perceived motor competence, F(2, 260) = 33.19, p =.001, ηp² =.20, indicating a large effect of the ACT intervention on changes in perceived motor competence over time. Similarly, significant interaction effects were found for actual motor competence across all tasks: heel-to-toe walking, F(2, 260) = 18.60, p =.001, ηp² =.12; walking/running, F(2, 260) = 16.55, p =.001, ηp² =.11; placing on plate, F(2, 260) = 31.31, p =.001, ηp² =.19; and building tower, F(2, 260) = 39.72, p =.001, ηp² =.23. Large interaction effects were observed across all tasks (ηp² ranging from.11 to.23). The intervention led to significant improvements over time in all motor competence tasks, with the most pronounced group differences observed in the placing on plate and building tower tasks, which demonstrated large effect sizes (ηp² =.11 and.23, respectively) (see Table 3).

**Fig 4:**
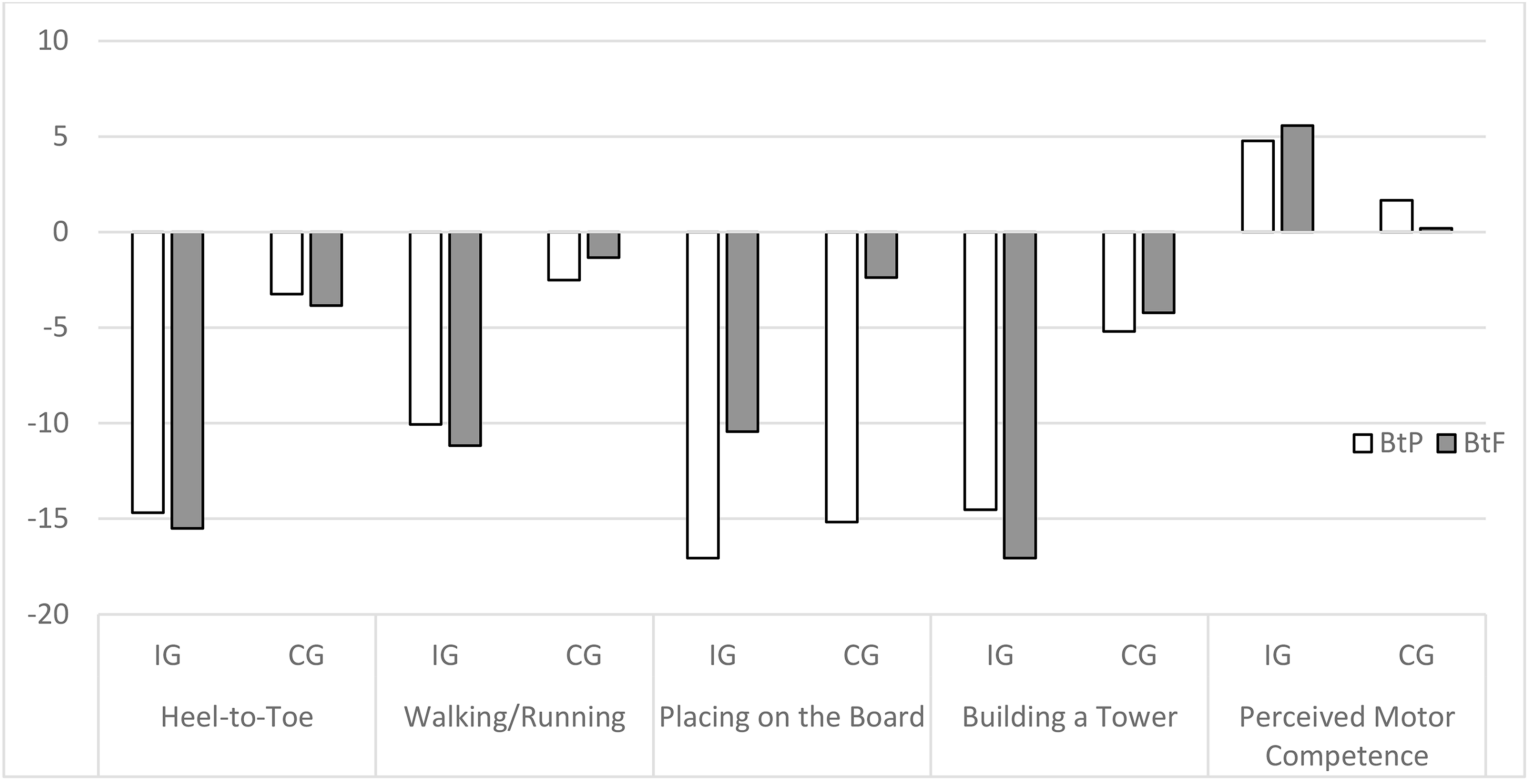
Percentage changes in perceived and actual motor competence by group from Baseline to Posttest (BtP) and Baseline to Follow-up (BtF).

Compared to the control group, the intervention group showed significantly greater improvements in perceived motor competence at both post-test (+4.78 vs. + 1.67; p = 0.02) and follow-up (+5.57 vs. + 0.19; p < 0.001). The intervention group showed significantly greater reductions in task completion times across all actual motor competence tasks compared to the control group. Specifically, performance improved on the Heel-to-Toe task at both post-test (14.68% vs. 3.25%; *p* =.002) and follow-up (15.5% vs. 3.85%; *p* <.001). Similar trends were observed in the Walking/Running task (10.05% and 11.18% vs. 2.5% and 1.33%; *p* =.004 and *p* <.001), Placing on the Board (17.05% and 10.44% vs. 15.16% and 2.37%; both *p* <.001), and Building a Tower (14.52% and 17.05% vs. 5.2% and 4.22%; both *p* <.001) (Fig 4).

## Discussion

This study demonstrated that Acceptance and Commitment Therapy (ACT) significantly improved motivational profiles, physical activity participation, physical performance, and both perceived and actual motor competence among adolescent girls with initially low activity levels. These results indicate that the ACT-based intervention was associated with improvements in self-determined motivation, physical activity participation, and motor outcomes. While ACT theory posits that such effects may operate through enhanced psychological flexibility, this construct was not measured in the current study; thus, no empirical claim about its role can be made.

A key finding was the improvement in motivational profiles, characterized by increases in intrinsic motivation, identified regulation, and autonomy, alongside decreases in external regulation and amotivation. This shift toward more self-determined motivation aligns with Self-Determination Theory (SDT), which emphasizes the role of autonomy, competence, and relatedness in internalizing motivation [11,32]. According to ACT theory, value clarification and acceptance-based strategies are intended to support engagement in meaningful behaviors, such as physical activity, even in the presence of discomfort. This theoretical mechanism—psychological flexibility—is proposed to complement SDT by facilitating autonomous action. However, because psychological flexibility was not assessed in this study, we cannot confirm whether this pathway was activated [33]. This likely helped participants reframe physical activity as personally meaningful rather than externally imposed. Previous research supports ACT’s effectiveness in promoting value-congruent behavior and reducing avoidance in adolescents [34,35]. It should be noted that psychological flexibility was not directly measured in this study, and its role is discussed here as a theoretical interpretation rather than an empirically confirmed mechanism.

Notably, introjected regulation—motivation driven by guilt, pressure, or conditional self-worth—remained unchanged, consistent with SDT findings that this type of motivation is more resistant to change due to its roots in conditional self-acceptance and contingent esteem [36,37]. Its persistence may reflect deeply ingrained patterns related to social comparison, perfectionism, and unmet emotional needs. Addressing these complex mechanisms may require integrating complementary strategies such as self-compassion or empathy-based interventions, which promote healthier internalization and reduce guilt-driven motivation [9].

The intervention also yielded significant improvements in physical activity participation and physical performance, corroborating evidence that ACT effectively supports health behavior change across diverse populations [13,15,38,39]. Unlike cognitive-behavioral approaches that seek to modify negative thoughts [40–42], ACT encourages acceptance of unpleasant thoughts and emotions while maintaining commitment to personally valued behaviors [18]. In this study, participants identified meaningful values—such as vitality, health, and family engagement—linking physical activity to broader life goals. This likely contributed to intrinsic motivation, enabling participants to overcome typical adolescent barriers such as low motivation, self-consciousness, and time constraints. These outcomes resonate strongly with SDT, which posits that autonomous motivation—grounded in personal values and self-endorsement—leads to more persistent and self-regulated engagement in behaviors [11,36]. Moreover, the results align with the Theory of Planned Behavior (TPB), emphasizing that intention to act is shaped by positive attitudes and perceived behavioral control [43]. ACT’s emphasis on values clarification and psychological flexibility may strengthen these constructs by fostering favorable attitudes toward physical activity, enhancing self-efficacy, and reinforcing long-term commitment. Moreover, ACT may counteract the typical decline in motivation for physical education during adolescence [44].

One notable outcome was the improvement in both perceived and actual motor competence, factors strongly linked to sustained physical activity and psychosocial benefits such as enhanced self-esteem and social acceptance [45,46]. Improving motor competence during adolescence can create a positive feedback loop, where increased skills lead to greater activity participation, further enhancing competence and psychological outcomes. The observed improvements in motor competence may reflect the influence of ACT’s emphasis on values-based action and present-moment engagement. While the intervention was designed to reduce performance-related avoidance and encourage persistence—consistent with ACT principles—these interpretations remain theoretical, as the study did not include measures of psychological flexibility, anxiety, or mindfulness [33].

Perceived competence is particularly important for intrinsic motivation and ongoing engagement without reliance on external rewards [47], consistent with SDT’s emphasis on autonomy-supportive environments that nurture competence and choice [48]. However, the study did not directly examine psychological flexibility as a mediator of changes in motor competence. Future research should clarify how enhancing psychological flexibility contributes to improvements in perceived and actual motor skills, and how these changes influence long-term physical activity behavior.

Our findings—that the ACT program enhanced autonomous motivation while reducing controlled motivation—resonate with recent conceptual work in motivational science. Ahmadi, Noetel (12) recently proposed a comprehensive classification of SDT-based motivational behaviors shown to effectively support basic psychological needs in educational and health settings. Notably, the ACT strategies employed in our intervention (e.g., linking movement to personal values, creating space for discomfort, emphasizing choice in activity selection) closely mirror several of these empirically supported practices, such as *providing meaningful rationales*, *acknowledging internal experiences*, and *encouraging self-endorsed action*. This alignment suggests that ACT may not only address cognitive and emotional barriers but also function as an implicit vehicle for delivering need-supportive motivational communication. Future studies could explicitly integrate Ahmadi et al.’s taxonomy into intervention design and process evaluation to clarify which specific motivational behaviors drive change [12].

### Strengths and limitations

This study’s strengths include its robust, theory-driven intervention based on Acceptance and Commitment Therapy, which specifically targets psychological processes that influence motivation and behavior. The randomized controlled design with longitudinal follow-up enhances the ability to infer causality and assess the sustainability of effects. Use of validated and diverse outcome measures—covering motivational profiles, physical activity participation, physical performance, and motor competence—provides a comprehensive evaluation of the intervention’s impact. Additionally, integrating both subjective self-report and objective performance assessments strengthens the validity of findings. However, several limitations constrain generalizability. The relatively small and demographically homogeneous sample restricts the generalizability of the results to broader populations. Reliance on self-reported data for some outcomes introduces the potential for response and social desirability biases. The lack of blinding may have introduced expectancy effects, potentially influencing participants’ responses and performance. Furthermore, the study did not include direct measurement of psychological flexibility directly, which restricts insight into mediating mechanisms. The follow-up period, while helpful, was not sufficiently long to determine the long-term maintenance of behavioral changes and fitness improvements. Lastly, given the limited number of schools (8 schools) in this study, it was not feasible to fully apply multilevel models to account for cluster effects, and this limitation may have slightly inflated or reduced the precision of the estimated intervention effects. Future research should aim to address these limitations by including more diverse samples, longer follow-up periods, objective physical activity measures (e.g., accelerometers), and controlling for additional covariates.

### Practical implications and future directions

This study suggests that integrating ACT-based methods into physical education could promote self-determined motivation and more consistent participation in physical activity. Training educators to model value-based reflection and acceptance of discomfort may support student autonomy and resilience. Future research should explore the long-term maintenance of ACT-related behavior change and examine moderating variables such as gender, cultural context, and baseline psychological flexibility. Combining ACT with other strategies, such as motivational interviewing or self-compassion interventions, may further strengthen outcomes—particularly in domains like introjected regulation.

## Conclusion

The results highlight the significant benefits of incorporating ACT strategies into physical education classes. ACT can enhance students’ intrinsic motivation and promote greater participation in physical activity. This approach supports not only improved physical performance and motor skills but also fosters a more positive and sustainable attitude toward exercise. Integrating ACT techniques into physical education curricula can empower teachers to create a supportive environment that encourages students to accept discomfort, focus on personal values, and develop lifelong healthy habits. Ultimately, this holistic method may improve students’ physical fitness, mental well-being, and long-term adherence to active lifestyles, helping to combat sedentary behavior and related health issues among youth.

## Supporting information

S1 Table Details of descriptive information about research variables. (DOCX)
