# Supplementary material for: Effects of a school-based ACT program on motivation, physical activity participation, motor competence, and physical performance in physically inactive female adolescent students
Source: PLoS One. 2026 Jun 10;21(6):e0343227. doi: 10.1371/journal.pone.0343227 (PMC13252786; doi:10.1371/journal.pone.0343227)
Supplement: S1 Table — (DOCX) [file pone.0343227.s001.docx]

| **Group Statistics** | | | | | |
| --- | --- | --- | --- | --- | --- |
|  | group | N | Mean | Std. Deviation | Std. Error Mean |
| Age | control | 66 | 15.5000 | 1.72091 | .21183 |
|  | expriment | 66 | 15.5000 | 1.72091 | .21183 |
| Weigth | control | 66 | 55.0303 | 9.22366 | 1.13535 |
|  | expriment | 66 | 54.5303 | 9.74635 | 1.19969 |
| bmi | control | 66 | 21.8049 | 2.64542 | .32563 |
|  | expriment | 66 | 21.4904 | 3.26692 | .40213 |
| Height | control | 66 | 158.4394 | 6.14223 | .75606 |
|  | expriment | 66 | 159.1061 | 6.67977 | .82222 |
| PAQA | control | 66 | 1.9482 | .40200 | .04948 |
|  | expriment | 66 | 1.9885 | .43874 | .05400 |
| PAQApost | control | 66 | 1.9432 | .45023 | .05542 |
|  | expriment | 66 | 2.3303 | .48370 | .05954 |
| PAQAfollowup | control | 66 | 1.9853 | .44284 | .05451 |
|  | expriment | 66 | 2.4250 | .55841 | .06873 |
| BREQ_amotivat_PRE | control | 66 | .8962 | .64791 | .07975 |
|  | expriment | 66 | .9470 | .67868 | .08354 |
| BREQ_amotivat_POST | control | 66 | .7083 | .57610 | .07091 |
|  | expriment | 66 | .4205 | .38769 | .04772 |
| BREQ_amotivat_Folow | control | 66 | .7462 | .54507 | .06709 |
|  | expriment | 66 | .4432 | .42921 | .05283 |
| BREQ_external_PRE | control | 66 | .9568 | .72201 | .08887 |
|  | expriment | 66 | .8850 | .74769 | .09203 |
| BREQ_external_POST | control | 66 | .7252 | .68426 | .08423 |
|  | expriment | 66 | .4452 | .47114 | .05799 |
| BREQ_external_Folow | control | 66 | .7202 | .67851 | .08352 |
|  | expriment | 66 | .4856 | .48280 | .05943 |
| BREQ_introject_PRE | control | 66 | 1.8264 | .88593 | .10905 |
|  | expriment | 66 | 1.6268 | .84316 | .10379 |
| BREQ_introject_POST | control | 66 | 1.7118 | .75798 | .09330 |
|  | expriment | 66 | 1.3832 | .94499 | .11632 |
| BREQ_introject_Folow | control | 66 | 1.7067 | .76001 | .09355 |
|  | expriment | 66 | 1.3882 | .94380 | .11617 |
| BREQ_identified_PRE | control | 66 | 2.6061 | .77591 | .09551 |
|  | expriment | 66 | 2.5379 | .63586 | .07827 |
| BREQ_identified_POST | control | 66 | 2.6439 | .78454 | .09657 |
|  | expriment | 66 | 2.7992 | .62821 | .07733 |
| BREQ_identified_Folow | control | 66 | 2.5833 | .80424 | .09899 |
|  | expriment | 66 | 2.8636 | .62662 | .07713 |
| BREQ_internal_PRE | control | 66 | 2.7879 | .65080 | .08011 |
|  | expriment | 66 | 2.8902 | .61100 | .07521 |
| BREQ_internal_POST | control | 66 | 2.8358 | .64849 | .07982 |
|  | expriment | 66 | 3.3485 | .59949 | .07379 |
| BREQ_internal_Folow | control | 66 | 2.8206 | .65623 | .08078 |
|  | expriment | 66 | 3.3674 | .61896 | .07619 |
| AMCQ_PRE | control | 66 | 88.8788 | 6.08786 | .74936 |
|  | expriment | 66 | 88.2273 | 5.57686 | .68646 |
| AMCQ_POST | control | 66 | 90.3485 | 5.72845 | .70512 |
|  | expriment | 66 | 92.4545 | 4.80122 | .59099 |
| AMCQ_Folow | control | 66 | 89.0455 | 5.58413 | .68736 |
|  | expriment | 66 | 93.1818 | 4.68355 | .57651 |
| CooperPRE | control | 66 | 996.5909 | 150.27738 | 18.49787 |
|  | expriment | 66 | 952.0152 | 106.95153 | 13.16482 |
| CooperPOST | control | 66 | 1031.8485 | 129.32800 | 15.91918 |
|  | expriment | 66 | 1131.0303 | 94.94419 | 11.68682 |
| CooperFollow | control | 66 | 1038.2121 | 127.98720 | 15.75414 |
|  | expriment | 66 | 1177.1515 | 93.02559 | 11.45066 |
| FlexibilityPRE | control | 66 | 26.0303 | 6.49253 | .79917 |
|  | expriment | 66 | 24.9242 | 6.00336 | .73896 |
| FlexibilityPOST | control | 66 | 27.7121 | 5.67251 | .69824 |
|  | expriment | 66 | 29.5606 | 5.18913 | .63874 |
| FlexibilityFollow | control | 66 | 27.7879 | 5.36517 | .66041 |
|  | expriment | 66 | 30.5758 | 5.18002 | .63762 |
| JumpPRE | control | 66 | 152.9242 | 18.51512 | 2.27905 |
|  | expriment | 66 | 150.8788 | 14.80178 | 1.82197 |
| JumpPOST | control | 66 | 155.8182 | 18.94032 | 2.33139 |
|  | expriment | 66 | 160.7121 | 17.00386 | 2.09303 |
| JumpFollowe | control | 66 | 156.1818 | 18.85076 | 2.32037 |
|  | expriment | 66 | 162.5000 | 16.43846 | 2.02343 |
| Run20PRE | control | 66 | 4.4794 | .60522 | .07450 |
|  | expriment | 66 | 4.6592 | .50192 | .06178 |
| Run20POST | control | 66 | 4.4565 | .57170 | .07037 |
|  | expriment | 66 | 4.0592 | .55010 | .06771 |
| Run20follow | control | 66 | 4.4837 | .53321 | .06563 |
|  | expriment | 66 | 4.0673 | .49016 | .06033 |
| HeeltoePRE | control | 66 | 10.5482 | 2.02921 | .24978 |
|  | expriment | 66 | 10.9074 | 2.00104 | .24631 |
| HeeltoePOST | control | 66 | 10.1942 | 1.84752 | .22741 |
|  | expriment | 66 | 9.3050 | 1.42011 | .17480 |
| Heeltoefollow | control | 66 | 10.1404 | 1.74237 | .21447 |
|  | expriment | 66 | 9.1974 | 1.47413 | .18145 |
| Run8PRE | control | 66 | 6.0003 | .54416 | .06698 |
|  | expriment | 66 | 6.1744 | .60775 | .07481 |
| Run8POST | control | 66 | 5.8559 | .62154 | .07651 |
|  | expriment | 66 | 5.5527 | .56652 | .06973 |
| Run8followe | control | 66 | 5.9215 | .63501 | .07816 |
|  | expriment | 66 | 5.4829 | .48427 | .05961 |
| PlatePRE | control | 66 | 23.8395 | 3.93887 | .48484 |
|  | expriment | 66 | 23.4292 | 3.92433 | .48305 |
| PlatePOST | control | 66 | 23.1752 | 3.82280 | .47055 |
|  | expriment | 66 | 20.6468 | 3.62989 | .44681 |
| PlateFollow | control | 66 | 23.3759 | 3.92783 | .48348 |
|  | expriment | 66 | 20.6347 | 3.43409 | .42271 |
| TowerPRE | control | 66 | 13.3415 | 2.93324 | .36106 |
|  | expriment | 66 | 13.2895 | 2.62185 | .32273 |
| TowerPOST | control | 66 | 12.8347 | 3.00724 | .37017 |
|  | expriment | 66 | 11.1923 | 2.35972 | .29046 |
| TowerFollow | control | 66 | 13.0347 | 2.98196 | .36705 |
|  | expriment | 66 | 11.0202 | 2.28039 | .28070 |
| ghad | control | 66 | 1.5844 | .06142 | .00756 |
|  | expriment | 66 | 1.5911 | .06680 | .00822 |
| Education | control | 66 | 9.5000 | 1.72091 | .21183 |
|  | expriment | 66 | 9.5000 | 1.72091 | .21183 |
| RAI_pre_Amotivate | control | 66 | -2.6886 | 1.94374 | .23926 |
|  | expriment | 66 | -2.8409 | 2.03604 | .25062 |
| RAI_post_Amotivate | control | 66 | -2.1250 | 1.72830 | .21274 |
|  | expriment | 66 | -1.2614 | 1.16308 | .14317 |
| RAI_follow_Amotivate | control | 66 | -2.2386 | 1.63521 | .20128 |
|  | expriment | 66 | -1.3295 | 1.28763 | .15850 |
| RAI_pre_external | control | 66 | -1.9136 | 1.44402 | .17775 |
|  | expriment | 66 | -1.7700 | 1.49537 | .18407 |
| RAI_post_external | control | 66 | -1.4503 | 1.36852 | .16845 |
|  | expriment | 66 | -.8903 | .94227 | .11599 |
| RAI_follow_external | control | 66 | -1.4403 | 1.35701 | .16704 |
|  | expriment | 66 | -.9712 | .96560 | .11886 |
| RAI_pre_introject | control | 66 | -1.8264 | .88593 | .10905 |
|  | expriment | 66 | -1.6268 | .84316 | .10379 |
| RAI_post_introject | control | 66 | -1.7118 | .75798 | .09330 |
|  | expriment | 66 | -1.3832 | .94499 | .11632 |
| RAI_follow_introject | control | 66 | -1.7067 | .76001 | .09355 |
|  | expriment | 66 | -1.3882 | .94380 | .11617 |
| RAI_pre_identified | control | 66 | 5.2121 | 1.55182 | .19102 |
|  | expriment | 66 | 5.0758 | 1.27171 | .15654 |
| RAI_post_identified | control | 66 | 5.2879 | 1.56908 | .19314 |
|  | expriment | 66 | 5.5985 | 1.25643 | .15466 |
| RAI_follow_identified | control | 66 | 5.1667 | 1.60847 | .19799 |
|  | expriment | 66 | 5.7273 | 1.25325 | .15426 |
| RAI_per_internal | control | 66 | 8.3636 | 1.95241 | .24032 |
|  | expriment | 66 | 8.6705 | 1.83301 | .22563 |
| RAI_post_internal | control | 66 | 8.5073 | 1.94548 | .23947 |
|  | expriment | 66 | 10.0455 | 1.79846 | .22137 |
| RAI_follow_internal | control | 66 | 8.4618 | 1.96869 | .24233 |
|  | expriment | 66 | 10.1023 | 1.85689 | .22857 |
| RAI_pre | control | 66 | 7.1471 | 4.24593 | .52264 |
|  | expriment | 66 | 7.5085 | 4.00489 | .49297 |
| RAI_post | control | 66 | 8.5080 | 4.02457 | .49539 |
|  | expriment | 66 | 12.1091 | 3.08760 | .38006 |
| RAI_follow | control | 66 | 8.2429 | 3.88626 | .47837 |
|  | expriment | 66 | 12.1406 | 3.28709 | .40461 |
| Standardized Residual for BREQ_amotivat_PRE | control | 66 | .0000 | .97655 | .12020 |
|  | expriment | 66 | .0000 | 1.02292 | .12591 |
| Standardized Residual for BREQ_amotivat_POST | control | 66 | .0000 | 1.17328 | .14442 |
|  | expriment | 66 | .0000 | .78957 | .09719 |
| Standardized Residual for BREQ_amotivat_Folow | control | 66 | .0000 | 1.11109 | .13677 |
|  | expriment | 66 | .0000 | .87492 | .10770 |
| Standardized Residual for BREQ_external_PRE | control | 66 | .0000 | .98238 | .12092 |
|  | expriment | 66 | .0000 | 1.01731 | .12522 |
| Standardized Residual for BREQ_external_POST | control | 66 | .0000 | 1.16481 | .14338 |
|  | expriment | 66 | .0000 | .80201 | .09872 |
| Standardized Residual for BREQ_external_Folow | control | 66 | .0000 | 1.15227 | .14184 |
|  | expriment | 66 | .0000 | .81992 | .10092 |
